# Cerebellar Resistance to Amyloid Plaque Deposition and Elevated Microglial ECM Proteoglycan Uptake in 5xFAD Mice

**DOI:** 10.3390/cells15020182

**Published:** 2026-01-19

**Authors:** Carla Cangalaya, Henning Peter Düsedau, Ildiko Rita Dunay, Alexander Dityatev, Stoyan Stoyanov

**Affiliations:** 1 Molecular Neuroplasticity Research Group, German Center for Neurodegenerative Diseases (DZNE), Leipziger Str. 44, Haus 64, D-39120 Magdeburg, Germany; carla-marcia.cangalaya-lira@dzne.de; 2 Institute of Inflammation and Neurodegeneration, Health Campus Immunology, Infectiology and Inflammation (GC-I3), Otto-von-Guericke-University, D-39120 Magdeburg, Germany; 3 Center for Behavioral Brain Sciences (CBBS), D-39106 Magdeburg, Germany; 4 Center for Intervention and Research on Adaptive and Maladaptive Brain Circuits Underlying Mental Health (C-I-R-C), German Center for Mental Health (DZPG), Halle-Jena-Magdeburg, D-07743 Jena, Germany; 5 Medical Faculty, Otto-von-Guericke-University, D-39120 Magdeburg, Germany

**Keywords:** extracellular matrix, perineuronal nets, proteoglycans, Alzheimer’s disease, microglia, cerebellum, cortex, synaptic pruning

## Abstract

In both Alzheimer’s disease (AD) patients and animal models, senile plaques are generally observed in the cerebral cortex rather than the cerebellum. The mechanisms underlying the regional resistance of the cerebellum to amyloid plaque deposition remain poorly understood. We investigated this cerebellar resistance using 5xFAD mice, an amyloidosis model with high expression of mutant human *APP* and *PSEN1* in the cortex and cerebellum. In aged 5xFAD mice, the cerebellum had minimal amyloid-β (Aβ) deposition despite robust transgene expression, correlating with lower expression levels of IBA1, CD68, TREM2, and CD36 (although elevated expression of CD45 and MHC I) compared to the cortex. Consistent with the absence of plaques, cerebellar tissue lacked the dystrophic VGLUT1-positive synaptic accumulations prominent in the cortex. Cerebellar microglia maintained a distinct, less inflammatory phenotype yet displayed efficient clearance activity. Notably, ASC inflammasome specks—capable of seeding Aβ aggregation—were paradoxically more abundant in the cerebellum, implying that rapid Aβ clearance prevents these seeds from driving plaque formation. Furthermore, key extracellular matrix (ECM) proteoglycans brevican and aggrecan were elevated in the 5xFAD cerebellum. Cerebellar microglia showed enhanced internalization of brevican alongside small Aβ aggregates, exceeding that in cortical microglia. These findings indicate that region-specific microglial and ECM interactions—particularly efficient uptake and degradation of ECM–Aβ co-aggregates—may underlie the cerebellum’s resilience to amyloid plaque pathology.

## 1. Introduction

Alzheimer’s disease (AD) represents the leading cause of dementia in the elderly and is characterized by a specific profile of neuropathological hallmarks. These include neural tissue loss, intracellular neurofibrillary tangles formed by hyperphosphorylated tau, and extracellular neuritic plaques containing aggregated amyloid-β (Aβ) peptides in critical brain regions such as the basal forebrain, hippocampus, and neocortex. In contrast, areas like the striatum and cerebellum are typically spared from this neurodegeneration [1,2]. Specifically, the cerebellum’s role in AD is often overlooked because it is typically one of the last brain regions in patients to accumulate Aβ deposits, which usually manifest as diffuse plaques [3].

Genetically, AD is classified into two forms: early-onset and late-onset. While mutations in genes like amyloid precursor protein (*APP*) and presenilin (*PSEN1/PSEN2*) are linked to the early-onset form of the disease, which is inherited in an autosomal dominant pattern, the genetics of late-onset AD is more complex. Late-onset AD, which is the most common form of the disease, is not caused by a single gene but is instead influenced by a combination of genetic, lifestyle, and environmental factors [4]. These mutations support the theory that the Aβ peptide plays a central role in AD. The Aβ peptide is a normal byproduct of cell metabolism, formed when the APP is cleaved by β-secretase and γ-secretase enzymes [5,6,7]. To investigate AD, scientists frequently employ transgenic mouse models that overexpress mutant amyloid precursor protein (APP) to simulate human pathology. A prominent example is the 5xFAD mouse model, which carries five familial AD mutations—three within the *APP* gene and two within the *PSEN1* gene. These mutations drive the overproduction of Aβ peptides, resulting in the onset of amyloid deposition as early as 1.5 months and subsequent memory impairment by 4–6 months of age. While a profound Aβ plaque burden is established in the somatosensory cortex, thalamus, and hippocampus by nine months, the amygdala and cerebellum remain notably resistant, exhibiting only minimal Aβ accumulation [8].

The mechanisms underlying the cerebellum’s relative resistance to amyloid pathology remain a critical area of investigation. Research links this resistance to microglia, the brain’s resident immune cells, given their established role in AD pathology [9,10]. For example, microglia-derived ASC specks, a multiprotein complex within microglia, cross-seed Aβ in Alzheimer’s disease [11], supporting the established link between inflammasome activation and the initiation and spreading of Aβ pathology. Microglia that adhere to Aβ plaques acquire a specific transcriptional signature known as “disease-associated microglia” (DAM). This signature largely emanates from the CD36, TREM2–DAP12 receptor complex, which transmits intracellular signals through the protein tyrosine kinase SYK. TREM2 receptor drives the microglial response to Aβ via both SYK-dependent and -independent pathways [12,13].

Emerging evidence suggests that the extracellular matrix (ECM)—a complex network of molecules providing structural and biochemical support to cells [14]—plays an important role in the etiology of AD together with microglia, both in its diffuse form and as perineuronal nets (PNNs). Components of the ECM, such as proteoglycans, can interact with Aβ and influence its aggregation and clearance [15]. Research indicates that PNN composition is significantly altered in AD; this impacts their detection by WFA lectin, leading to reduced quantification that correlates with plaque burden [16,17]. Furthermore, activated microglia in AD engulf damaged PNNs in both mice and humans. Notably, chronic microglial depletion prevents PNN loss despite persistent plaques, suggesting microglia actively contribute to PNN degradation [16]. In our previous work, in 5xFAD mice, we have shown that the migration of both microglia and myeloid cells is disrupted during the initial stages of amyloidosis; however, this motility can be partially restored through ECM attenuation via chondroitinase ABC (ChABC) treatment [18].

The spatial heterogeneity of amyloid pathology in AD is a prominent feature, with the cerebellum consistently demonstrating a notably lower plaque burden compared to other brain regions. To elucidate this regional resilience, here we investigated the influence of two key factors that also exhibit regional variation: microglia and the ECM. We hypothesize that differences in the activation state and function of microglia, as well as specific characteristics of the ECM, contribute to the cerebellum’s relative protection against amyloid accumulation. In this study, we focused on microglial expression profiles of three main cortical regions and cerebellum in 5xFAD transgenic mice and wild-type mice at 12 months of age. Our data provide intriguing insights into the factors and pathways implicated in the regional differences of late-onset pathogenesis of AD.

## 2. Materials and Methods

### 2.1. Experimental Animals

Male 5xFAD (5xFAD^+/−^.Bl6) mice (strain: B6.CgTg [APPSwFlLon, PSEN1* M146L* L286V] 6799Vas/Mmj-ax, Jackson Laboratories, Bar Harbor, Maine, USA, #34848-JAX) at 12 months of age were used to study late-stage AD pathology. At 12 months of age, these mice exhibit significant amyloid burden, which initially appears at 2 months in the subi-culum and deep cortex. The genetic background includes five human mutations (three in *APP* and two in *PSEN1*) expressed under the neuron-specific Thy1 promoter [19]. Environmental conditions were standardized (12-h light cycle, with water and food ad libitum), and all research was conducted under license 42502-2-1346 DZNE, as approved by the Saxony-Anhalt Ethical Committee in alignment with German animal welfare legislation.

### 2.2. Cell Isolation

Brains from 12-month-old mice (a total of six 5xFAD and six WT mice) were harvested following deep anesthesia with a ketamine/xylazine mixture (90/18 mg/kg) in 0.9% NaCl and transcardial perfusion using sterile PBS. Utilizing the Allen Mouse Brain Atlas for anatomical guidance [20], the cerebellum and cortex were isolated and homogenized in a specialized buffer (HBSS supplemented with 13 mM HEPES and 0.68% glucose). The resulting tissue suspension was filtered through a 70 μm cell strainer. Immune cells were isolated by centrifuging the homogenate using a 30–70% discontinuous Percoll gradient (GE Healthcare, Chicago, IL, USA) with the target population recovered from the 30/70% Percoll interphase. Following two wash steps, the isolated cells were processed for flow cytometric analysis.

### 2.3. RNA Isolation and Reverse Transcription qPCR

Brain samples were extracted and dissected into cortex and cerebellum as described above. Tissue samples were then stored in RNAlater solution (Sigma-Aldrich, St. Louis, MO, USA, #R0901) before RNA isolation. The tissue was homogenized in a lysis buffer using BashingBeads Lysis tubes (Zymo Research Europe, Freiburg, Germany), and RNA was isolated using an AllPrep DNA/RNA Mini Kit (Qiagen, Hilden, Germany). The concentration and purity of the RNA were measured using a NanoDrop 2000 spectrophotometer (Thermo Fisher, Waltham, MA, USA), and RNA was stored at −80 °C until further use. Gene expression levels were evaluated using reverse transcription quantitative PCR (RT-qPCR). To this end, 30 ng of isolated RNA was amplified with a TaqMan^®^ RNA-to-CT™ 1-Step Kit (Applied Biosystems, Foster City, CA, USA) [21]. Using a LightCycler^®^ 96 (Roche, Basel, Switzerland), the thermal-cycling process included a reverse transcription at 48 °C for 30 min, followed by a 10-min enzyme activation phase at 95 °C. This was followed by 55 cycles of denaturation at 95 °C for 15 s and annealing/extension at 60 °C for one minute. Relative mRNA levels were quantified using *Hprt* as a reference gene, calculated as the ratio of the target gene to the reference gene, and normalized to the mean values of the WT group, with reference gene validation performed as previously described [21,22].

### 2.4. Flow Cytometry

To analyze cell phenotypes using flow cytometry, freshly isolated cells were first stained with a fixable Zombie NIR dye to distinguish between live and dead cells (BioLegend, San Diego, CA, USA). An anti-FcγIII/II receptor antibody was added to prevent unspecific binding of antibodies. The cells were then stained with a panel of fluorochrome-conjugated antibodies targeting specific cell surface markers in FACS buffer (PBS containing 2% fetal bovine serum and 0.1% sodium azide). These antibodies included: FITC-MHC Class I (clone 28-14-8), APC-CD11b (clone M1/70) (purchased from eBioscience™, San Diego, CA, USA), and Brilliant Violet™ 510-CD45 (clone 30-F11) (purchased from BioLegend, San Diego, CA, USA). Cells were acquired using an Attune NxT flow cytometer (Thermo Fisher, Waltham, MA, USA). The collected data were then analyzed with FlowJo software (version 10.5.3, FlowJo LLC, Ashland, OR, USA). To account for autofluorescence and establish correct gating, Fluorescence Minus One (FMO) controls were used.

### 2.5. Immunohistochemistry and Imaging

A total of three 5xFAD and three WT mice were deeply anesthetized using a solution of ketamine (90 mg/kg) and xylazine (18 mg/kg) in 0.9% NaCl. They were then transcardially perfused with a 4% paraformaldehyde (PFA) solution in phosphate-buffered saline (PBS). The dissected brains were incubated in the same PFA/PBS solution and cryoprotected in a 30% sucrose solution (in phosphate buffer) for 48 h, and then frozen in 100% 2-methylbutane at −80 °C. Sagittal sections, 50-µm-thick, were cut using a cryostat. The resulting cortical and cerebellar sections were kept in a storage solution (one part ethylene glycol, one part glycerin, and two parts PBS at pH 7.2) at 4 °C for later processing.

For immunostaining, the free-floating sections were first permeabilized with 0.5% Triton X-100 in phosphate buffer (PB) for 10 min at room temperature (RT). This was followed by a 1-h incubation at RT in a blocking solution (PB supplemented with 0.3% Triton X-100 and 5% normal goat serum). The sections were then treated with the primary antibody (mouse anti-human Amyloid-beta-N-82E1, IBL, Minneapolis, MN, USA, #10303, 1:250; rabbit anti-ASC, Adipogen, San Diego, CA, USA, #AG-25B-0006TS-C100, 1:200; rabbit anti-IBA1, Wako, Richmond, VA, USA, #019-19741, 1:500; rat anti-CD68, Bio-Rad, Hercules, CA, USA, #MCA1957, 1:500; chicken anti-VGLUT1, Synaptic Systems, Göttingen, Germany, #135316, 1:1000; rabbit anti-aggrecan, Merck Millipore, Darmstadt, Germany, #AB1031, 1:1000; guinea pig anti-brevican, John et al., 2006 [23]) for 48 h at 4 °C. After three washes in PB (10 min each at RT), the sections were incubated with a conjugated secondary antibody on a shaker for three hours at RT. Finally, the brain sections were washed, mounted with a fluoromount reagent (Sigma, Darmstadt, Germany, #F4680), and imaged using a Zeiss, Jena, Germany, LSM 700 microscope at 5×, 20×, and 63× magnifications. Using the Franklin and Paxinos (2008) [24] mouse brain atlas, we identified specific coordinates for each region in sagittal sections to ensure reproducibility and precision. Stereotactic coordinates for the targeted brain regions were defined as follows: the motor cortex (MC) was delineated at ML 2.04 mm, with an AP range of 2.8 to 1.0 mm and a DV depth of 0.5 to 2.0 mm. The somatosensory cortex (SC) was identified at ML 2.04 mm (AP 1.5 to −1.5 mm; DV 0.0 to 1.2 mm), while the retrosplenial cortex (RSC) was localized to ML 0.48 mm, spanning AP −1.0 to −4.0 mm and DV 0 to 1.0 mm. Within the cerebellum, the deep cerebellar nuclei (DCN) were targeted at approximately ML 2.04 mm, AP −6.0 mm, and DV 3.0 mm. The overlying cerebellar cortex (CC) was mapped within the coordinates of ML 2.04 mm, AP −5.0 to −8.0 mm, and DV 1.0 to 3.0 mm.

### 2.6. Histochemical Measurements

To evaluate amyloid plaques (labeled with Aβ antibody) across the entire brain hemisphere, we captured large images using a 5× objective (NA 0.2). Microscope settings were kept constant for the acquisition of 4–6 z-stacks per image. Using the FIJI/ImageJ 1.53c platform, we generated maximum intensity projections from these stacks and manually delineated the cerebral hemisphere with the polygon selection tool for further analysis.

To quantify the immunoreactivity of ASC and Aβ, we captured confocal images with a 63× objective on comparable brain sections from each animal. Maximum intensity projections were generated from six z-stack images using FIJI/ImageJ software. We then used the Otsu dark automatic thresholding method in FIJI/ImageJ 1.53c to analyze the percentage of the immunoreactive area for both ASC and Aβ.

To quantify the microglial IBA1 area and cell count, we acquired three 20× images from different cortical and cerebellar regions for each mouse. We processed these images using FIJI/ImageJ. First, we converted the z-series stacks into maximum intensity projections. Then, we automatically thresholded the IBA1 staining with the Otsu dark method. Finally, we used the “Particle Analysis” tool to determine the percentage of IBA1+ area and count the number of microglial cells.

We used z-stack images to blindly quantify microglial engulfment. To create a microglia mask, we first applied a consistent threshold determined from the entire stack’s histogram. We then used an unsharp mask filter to enhance microglial features, followed by a despeckle step to remove noise. A custom ImageJ macro was used to generate a profile of colocalized pixels between CD68 and VGLUT1 using Otsu’s method and color thresholding in the HSV color space. Finally, for automated 3D colocalization and quantification per microglia cell, we used both the ImageJ 1.53c 3D object counter plugin and the 3D viewer in Zen software 3.2.

To quantify dystrophic neurites (VGLUT1) in various brain regions, a consistent manual intensity threshold for VGLUT1 was applied to all sections. After this thresholding step, we analyzed the resulting particles to record their relative area (%), number, and average size.

We used high-resolution microscopy to capture images of brevican and aggrecan staining. We measured the intensity of both brevican and aggrecan in specific brain regions using a confocal microscope with a 63× objective on comparable sections from each animal. The mean intensity for these antibodies was quantified using FIJI.

To quantify Aβ+brevican puncta within microglial (IBA1+) masks, we obtained 4–6 z-stacks using a 63× oil immersion objective (NA 1.4). We kept the microscope settings consistent for all samples. The number of Aβ+brevican puncta was automatically counted using the FIJI Synapse Counter plugin.

### 2.7. Statistical Analysis

We used unpaired *t*-tests for comparisons between two groups. For multiple treatment groups, we performed a two-way ANOVA followed by the indicated post hoc tests. A *p*-value of less than 0.05 was considered statistically significant. All analyses were conducted using GraphPad Prism^®^ (Version 7.0, GraphPad Software, San Diego, CA, USA), and specific statistical details and *p*-values can be found in the figure legends. Statistical significance was determined using GraphPad Prism, where *p* values are represented as follows: * *p* ≤ 0.05, ** *p* ≤ 0.01, *** *p* ≤ 0.001, and **** *p* ≤ 0.0001, with *p* > 0.05 considered non-significant (ns).

## 3. Results

### 3.1. Differential Amyloid-Beta Accumulation in Cortical and Cerebellar Regions

**Figure 1:**
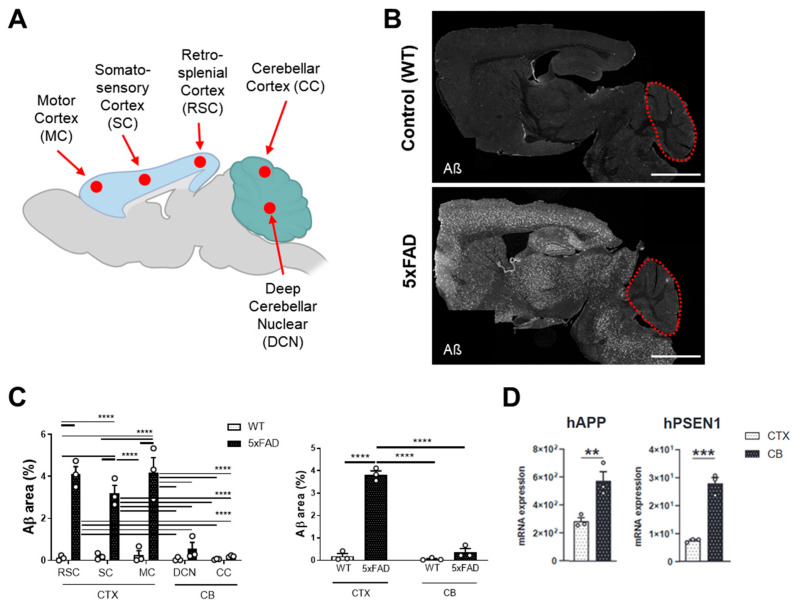
Accumulation of Aβ plaques in the cortex and cerebellum of 5xFAD mice. (**A**) Schematic illustration of the analyzed brain regions. The regions within the Cerebral Cortex (CTX) include the Motor Cortex (MC), Somatosensory Cortex (SC), and Retrosplenial Cortex (RSC). The regions within the Cerebellum (CB) include the Deep Cerebellar Nuclei (DCN) and the Cerebellar Cortex (CC). (**B**) Representative panoramic images of brain sections from WT and 5xFAD mice, stained with anti-Aβ. The red dashed line indicates the cerebellum in sagittal brain sections of WT and 5xFAD mice. Scale bar = 3 mm. (**C**) Quantification of the area percentage positive for Aβ in different brain regions in both WT and 5xFAD mice. (**D**) Gene expression levels of *hAPP* and *hPSEN1* in the cortex and cerebellum of WT and 5xFAD mice. ** *p* ≤ 0.01, *** *p* ≤ 0.001, and **** *p* ≤ 0.0001.

To study the spatial differences of Aβ deposition in a naturally aged murine AD model, we compared the Aβ immunopositive (Aβ+) area between brain regions in 12-month-old 5xFAD and WT mice. We detected a substantial increase in Aβ+ area in all cortical regions of 5xFAD when compared to WT mice (Figure 1B). The Aβ+ area percentage was markedly higher in cortical regions (RSC, SC, and MC) compared to cerebellar regions (DCN and CC) (3.84 ± 1.60% vs. 0.37 ± 0.15%, *p* < 0.0001) in 5xFAD mice (Figure 1C). Among all regions studied, the MC and RSC showed the most prominent Aβ accumulation.

qPCR analysis confirmed high expression of *hAPP* and *PSEN1* in the cortex of 5xFAD mice. Although amyloid plaque density in the cerebellum of 5xFAD mice is not distinguishable from that of the WT control, our RNA analysis revealed a significant increase in the expression of both *hAPP* and *hPSEN1* in the cerebellum of 5xFAD mice (Figure 1D).

**Figure 2:**
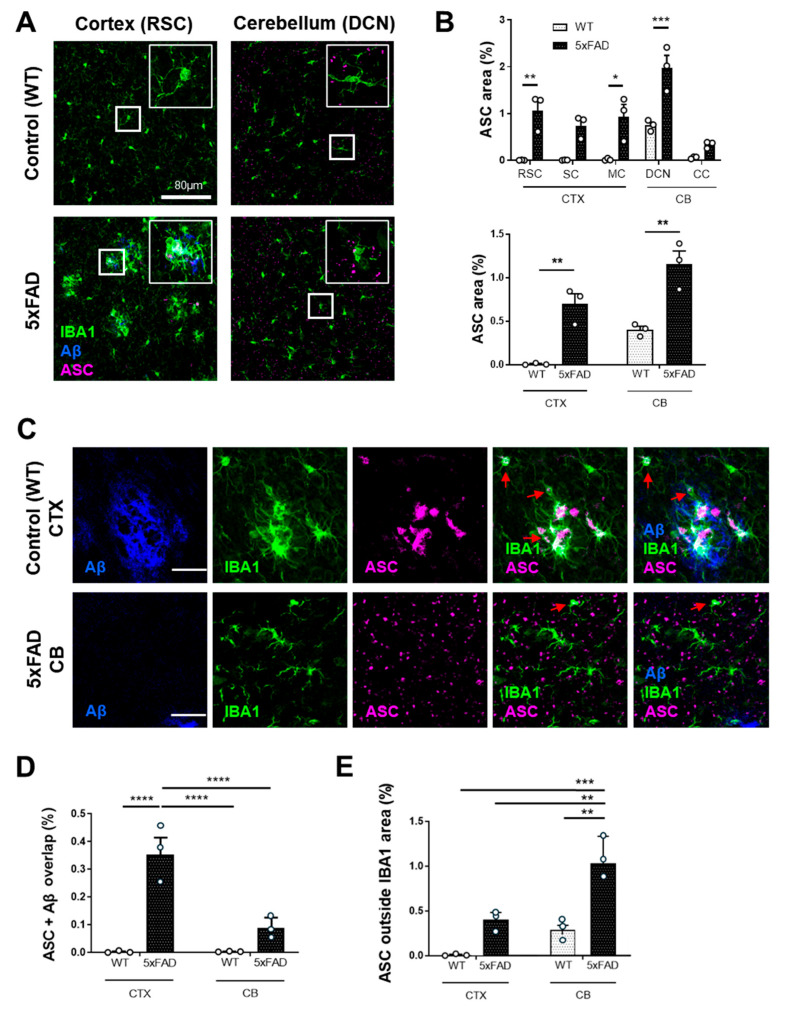
Accumulation of ASC in the cortex and cerebellum of 5xFAD mice. (**A**) Representative images of the cortex (RSC) and cerebellum (DCN) from WT and 5xFAD mice stained for the microglia marker IBA1, Aβ, and ASC. Scale bar = 80 µm. Magnified insets show IBA1 and ASC or Aβ colocalization within microglia. (**B**) Quantification of the area percentage positive for ASC in different brain regions in both WT and 5xFAD mice. (**C**) ASC expression within microglia in the cortex and cerebellum of 5xFAD mice. Red arrows indicate representative single microglia. Scale bar = 28 µm. (**D**,**E**) Quantification of the area percentage with ASC and Aβ colocalization, as well as the ASC-positive area outside of the IBA1-positive area, comparing cortical and cerebellar regions. * *p* ≤ 0.05, ** *p* ≤ 0.01, *** *p* ≤ 0.001, and **** *p* ≤ 0.0001.

Recently, microglia-released ASC specks have been shown to promote cross-seeding of Aβ, thereby accelerating plaque formation in mouse models of AD [11]. With regard to our initial findings, we hypothesized that ASC specks play a different role in the pathology of the cerebellum in 5xFAD mice. Indeed, our analysis confirmed previous reports and showed that ASC specks were more abundant in 5xFAD mice than in WT mice in both cortical and cerebellar regions (Figure 2A,B). However, no statistically significant difference was observed between the cerebellum and cortex of 5xFAD mice (1.16 ± 0.15% vs. 0.69 ± 0.12%, *p* = 0.07) (Figure 2B). Interestingly, in 5xFAD mice, ASC colocalized with Aβ plaques in the cortex (Figure 2C,D); however, upon closer inspection, we found that this high ASC-Aβ expression was due to plaque-associated microglia expressing ASC (Figure 2C). In contrast, in the cerebellum, ASC was localized outside the IBA1-positive area (Figure 2E), suggesting a predominantly non-microglial, extracellular pattern. This paradoxical finding further supports the notion that plaque formation in the AD mouse brain does not depend solely on ASC speck expression and hAPP/PSEN1, but may also depend on the balance between Aβ deposition and clearance. Thus, we wondered whether a more efficient clearance mechanism in the cerebellum could underlie our observation of a diminished seeding of Aβ plaques.

### 3.2. Microglia Responses to Aβ Plaques

As brain-resident macrophages, microglia perform a variety of tasks, including the removal of debris, dead cells, and *Aβ*. Given that cerebellar microglia are physiologically divergent from cortical populations, with potentially distinct roles in synaptic plasticity and pathogenesis [25], we investigated microglia characteristics in both brain regions in association with the marked differences in amyloid presence.

**Figure 3:**
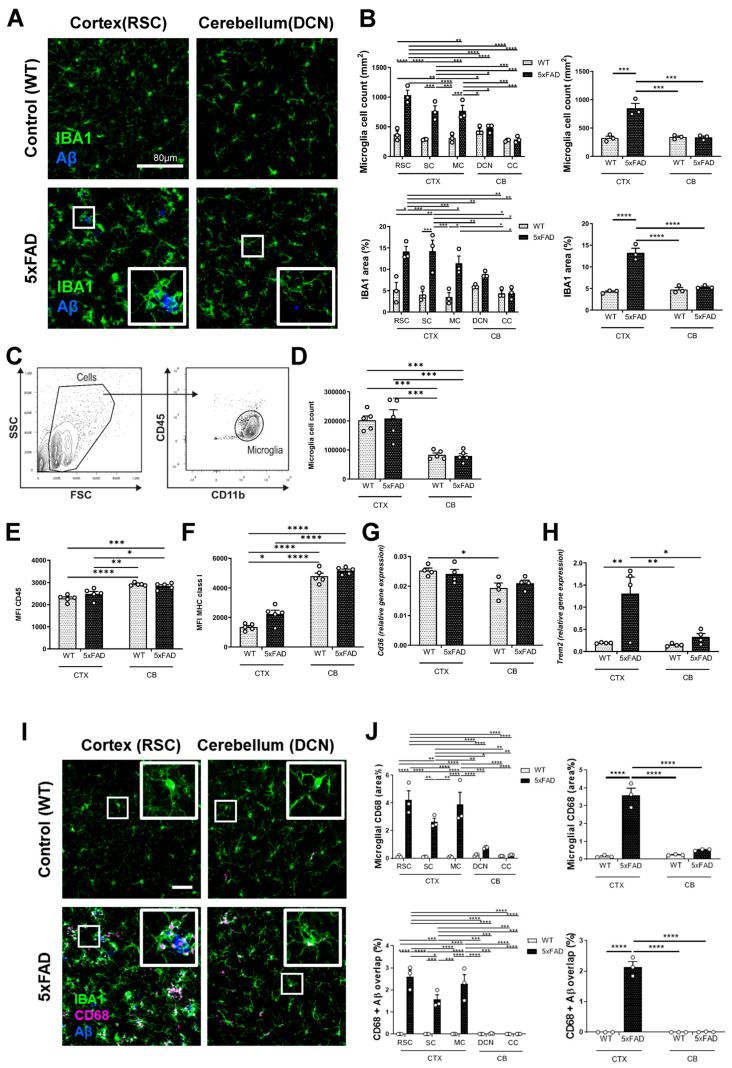
Microglia of different brain regions display different states in response to Aβ burden. (**A**) Representative images of RSC and DCN sections from WT and 5xFAD mice co-stained for IBA1 and Aβ. Scale bar = 80 µm. (**B**) Graphs showing the microglial cell count per square millimeter (mm^2^) and the percentage of the area covered by IBA1-positive cells (IBA1 area %) in different brain regions of WT and 5xFAD mice. (**C**) Flow cytometry plot illustrating the gating strategy used to identify microglia based on their size (FSC) and granularity (SSC), and by the expression of the cell surface markers CD45 and CD11b. (**D**) Quantification of microglia cell counts from flow cytometry in the cerebral cortex and cerebellum of WT and 5xFAD mice. (**E**,**F**) Graphs displaying the median fluorescence intensity (MFI) of CD45 and MHC class I on microglia from the cortex and cerebellum of WT and 5xFAD mice, serving as indicators of microglial activation. (**G**,**H**) The relative gene expression levels of CD36 (scavenger receptor) and TREM2 (gene associated with microglial function) in the cortex and cerebellum of WT and 5xFAD mice. (**I**) Representative images of the RSC and DCN from WT and 5xFAD mice, co-stained with IBA1, CD68, and Aβ. White colors denote the colocalization of three markers. Scale bar = 40 µm. (**J**) Graphs quantifying the percentage area covered by microglial CD68 and the percentage of overlap between CD68 and Aβ within microglia in different brain regions of WT and 5xFAD mice. * *p* ≤ 0.05, ** *p* ≤ 0.01, *** *p* ≤ 0.001, and **** *p* ≤ 0.0001.

In WT controls, we observed no difference in the relative density of microglia between regions. This, however, was changed in the context of AD pathology. In the cerebellum of 5xFAD mice, there was a lower microglial density compared to the cortex (Figure 3A,B). Specifically, the percentage of IBA1+ area was significantly higher in the cortex than in the cerebellum of 5xFAD mice (13.26 ± 1.05 vs. 5.34 ± 0.19; *p* < 0.0001) (Figure 3A,B). This suggests a strong microglial recruitment or activation in response to the higher Aβ burden in the cortex. To investigate the microglial response, key markers of inflammation were analyzed by flow cytometry and gene expression analysis (Figure 3C–H). In the cerebellum, we observed lower expression levels of Trem2 and CD36 compared to the cortex (Figure 3G,H). However, the surface expression of CD45 and MHC class I was elevated in the cerebellum (Figure 3E,F), indicating a different activation state of microglia. In this regard, immunofluorescence staining showed a notable increase in the CD68 signal, a lysosomal marker and proxy for ongoing phagocytosis, in the vicinity of Aβ plaques in the cortical regions of 5xFAD mice (Figure 3I,J). In 5xFAD mice, the percentage of microglial CD68 area was significantly higher in the cortical regions compared to the cerebellum (3.6 ± 0.39% vs. 0.52 ± 0.03%, *p* < 0.0001). Furthermore, the overlap between CD68 and Aβ was also significantly greater in the cortex than in the cerebellum (2.14 ± 0.18% vs. 0.01 ± 0.01%, *p* < 0.0001) (Figure 3F). In the cortex, high CD68 levels were observed alongside numerous amyloid plaques, suggesting a dysregulated microglial state. Conversely, the cerebellum showed lower CD68 expression, which correlated with other markers such as CD36 and TREM2. This suggests a more efficient mechanism of Aβ phagocytosis and indicates a dysregulation of microglial phagocytosis in the cortex, specifically in the vicinity of Aβ plaques.

### 3.3. Microglial Interactions with Neurons and Synaptic Integrity Differ Between Brain Regions

**Figure 4:**
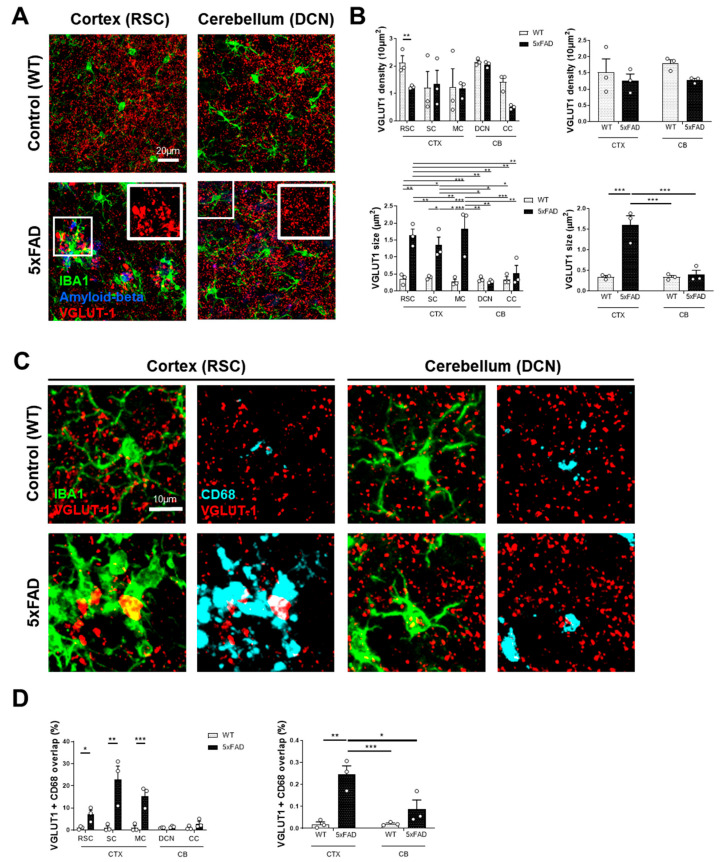
Microglial interactions with neurons and synaptic dystrophies differ between the cortex and the cerebellum. (**A**) Representative images of brain sections stained for IBA1, Aβ, and VGLUT1 from the cortex and cerebellum of WT and 5xFAD mice. The insets provide a magnified view of the selected areas. Scale bar = 20 µm. (**B**) Graphs showing the quantification of density and size of VGLUT1-positive puncta across different brain regions in WT and 5xFAD mice. (**C**) High-magnification images of the cortex and cerebellum, stained for IBA1, VGLUT1, and CD68 in WT and 5xFAD mice. The images illustrate the interaction between microglia, their phagocytic activity, and glutamatergic synapses (VGLUT1). Scale bar = 10 µm. (**D**) Graphs showing the percentage of overlap between VGLUT1 and CD68 signals within microglia across different brain regions in WT and 5xFAD mice. * *p* ≤ 0.05, ** *p* ≤ 0.01, and *** *p* ≤ 0.001.

Since microglial CD68 was only associated with amyloid plaques, and a dysregulation of microglial phagocytosis has been linked to increased pruning of synaptic terminals, we investigated whether microglia were phagocytosing synapses and if this aspect exhibited regional specificity in 5xFAD mice. While our analysis did not reveal a significant decrease in overall synaptic density (VGLUT1) in 5xFAD mice compared to WT mice across both cortical and cerebellar regions, the cerebellum was notably spared from the significant synaptic pathology observed in the cortex (Figure 4A). The cortex exhibited a significant increase in the VGLUT1+ puncta size relative to the cerebellum, which suggests the formation of dystrophic synaptic accumulations surrounding Aβ plaques (1.6 ± 0.22 μm^2^ vs. 0.4 ± 0.11 μm^2^, *p* = 0.0007) (Figure 4B). Furthermore, the overlap between VGLUT1 and CD68 was significantly higher in the cortex than in the cerebellum in 5xFAD mice (0.25 ± 0.04% vs. 0.09 ± 0.04%, *p* = 0.0212) (Figure 4C,D). However, this overlap was incomplete because the VGLUT1 aggregates did not completely localize within the CD68- or microglia-positive area. This data suggests that cortical microglia in 5xFAD mice appear to have a dysfunctional phagocytic state, as they associate with plaques and dystrophic synaptic accumulations but fail to fully engulf them (Figure 4C). In contrast, cerebellar microglia exhibit a preserved phagocytic capacity, as evidenced by fewer but complete engulfment events of Aβ and VGLUT1 (Figure 4C), further suggesting a neuroprotective environment within this brain region.

### 3.4. Extracellular Matrix Proteoglycan Clusters Facilitate Microglial Aβ Clearance in the Cerebellum

**Figure 5:**
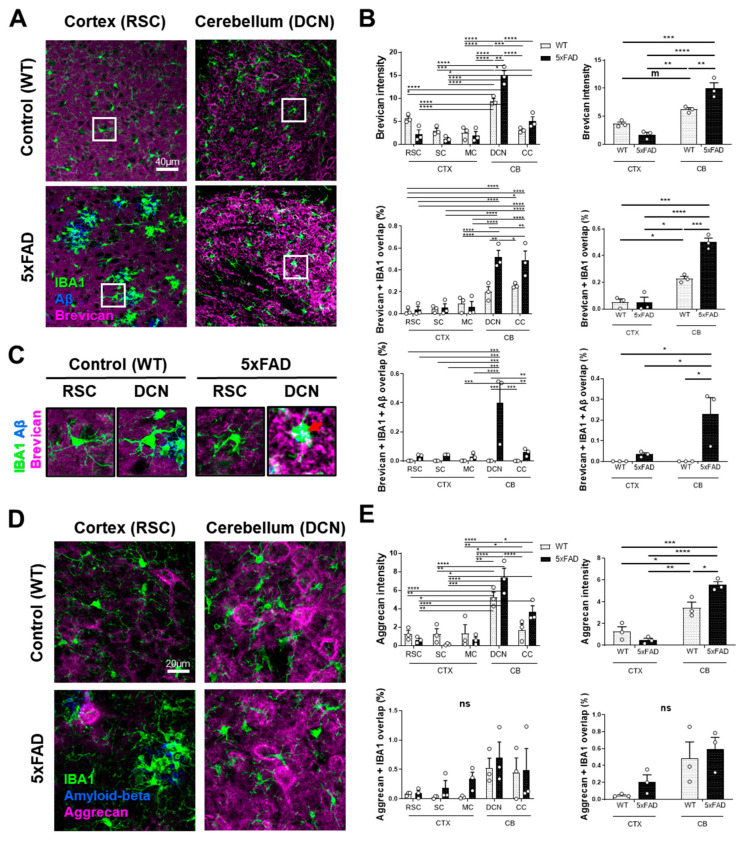
ECM proteoglycan clusters are related to microglial clearance of Aβ. (**A**) Representative images of sections stained for IBA1, Aβ, and brevican of the cortex and cerebellum from WT and 5xFAD mice. Scale bar = 40 µm. (**B**) Graphs quantifying brevican intensity and the overlap between brevican and IBA1-positive microglia, as well as the overlap between brevican, IBA1, and Aβ within microglia for different brain regions and for the broader CTX and CB areas. (**C**) High-magnification images (35 µm × 35 µm) showing clusters (red arrow) of Aβ and brevican in a microglial cell in the cortex and cerebellum of WT and 5xFAD mice. (**D**) Representative images of the cortex and cerebellum stained for IBA1, Aβ, and aggrecan. Scale bar = 20 µm. (**E**) Graphs quantifying the intensity of aggrecan and the overlap between aggrecan and IBA1-positive microglia for different brain regions and for the broader CTX and CB areas. m = marginal *p* = 0.05, * *p* ≤ 0.05, ** *p* ≤ 0.01, *** *p* ≤ 0.001, **** *p* ≤ 0.0001, and ns *p* > 0.05.

Recently, our work has highlighted the role of ECM proteins in mediating complement-dependent pathways of synapse removal [22]. Given the heterogeneous distribution of ECM-related proteins across different brain regions, we next explored the role of the ECM in the regional differences of amyloid deposits between the cortex and cerebellum by examining the expression of proteoglycans brevican and aggrecan. In line with our previous report, the cerebellum of WT mice displayed higher levels of brevican and aggrecan expression when compared to the cortex. Interestingly, in the cerebellum of 5xFAD mice, the increase in both brevican (9.96 ± 0.99 vs. 1.74 ± 0.41, *p* < 0.0001) (Figure 5A–C) and aggrecan expression (5.52 ± 0.31 vs. 0.47 ± 0.18) was even more pronounced (Figure 5D,E). Importantly, the overlap between brevican and IBA1 was markedly increased in the cerebellum when compared to the cortex (0.5 ± 0.03 vs. 0.05 ± 0.04, *p* < 0.0001) (Figure 5A,B). This suggests a strong interaction between microglia and brevican. Moreover, as shown in Supplementary Figure S1, the area of brevican/Aβ-positive puncta was significantly higher in the 5xFAD cerebellum compared with the cortex. Furthermore, our image analysis showed a higher percentage of brevican overlapping with microglia and Aβ (0.230 ± 0.08 vs. 0.034 ± 0.01, *p* = 0.0329) in the cerebellum (Figure 5C), suggesting that the cerebellar ECM-Aβ aggregates are more efficiently formed and internalized by microglia than the cortical ones.

## 4. Discussion

Our findings highlight that the cerebellum, despite expressing high levels of mutant human APP and PSEN1 genes, remains largely protected from amyloid plaque formation and synaptic pathology seen in the cortex. We propose that this neuroprotective environment is driven by a unique interplay between microglia and the ECM. Specifically, we found that cerebellar microglia, while not overtly inflammatory, maintain a preserved phagocytic capacity and efficiently clear ECM-Aβ co-aggregates. This contrasts with cortical microglia, which appear to have a dysregulated phagocytic state.

### 4.1. Regional Differences in Amyloid Pathology and Microglial Function

In line with our observations of lower Aβ accumulation in the cerebellum compared to the cortex in the 5xFAD mouse model, human studies have reported similar findings. There is an association between the delayed appearance or absence of Aβ plaques in the cerebellum and non-demented elderly individuals [26]. This suggests that cerebellar parenchymal amyloidosis has a later onset and a smaller impact on cerebellar structure and function. Furthermore, we detected positive immunoreactive spots in the cerebellar cortex and parenchyma, but not distinct plaques. This is also consistent with human studies where different techniques used to visualize amyloid plaques in fixed tissue could differentiate small deposits of Aβ. For example, silver staining can reveal a diffuse morphology of Aβ deposits in the cerebellum without distinct plaque formation [27]. A common pattern is a linear, hazy deposition of Aβ-42, which is thought to be along Bergmann glial fibers and is often perpendicular to the pial surface [26]. This unique pattern of Aβ-42 deposition in the cerebellum, when compared to other brain regions, may be attributed to its distinct anatomy and ECM composition, enhanced clearance of APP, or its late involvement in AD pathology, since studies have linked Aβ-42 deposition in the cerebellum to more advanced AD Braak stages [28].

The 5xFAD mouse model, which expresses mutant human APP and PSEN1, provides a robust system for studying familial AD and its regional variability. Consistent with previous findings [8,29], we observed that while Aβ plaques are prominent in the cortex, the cerebellum remains largely spared from significant amyloid accumulation. This is a crucial distinction, as our results show that both *APP* and *PSEN1* genes are highly expressed in both regions, indicating that the mere expression of these genes is not the sole determinant of pathology. Our study revealed another paradox: ASC inflammasome specks, which are known to seed Aβ aggregation [11], were more abundant in the cerebellum than in the cortex, but the cerebellum remains largely spared from significant amyloid accumulation. We found that ASC expression in the cerebellum was predominantly extracellular, whereas in the cortex, ASC colocalized more frequently with microglia—a phenomenon even more evident around amyloid plaques. This may suggest that in the cerebellum, ASC specks are released and remain in the extracellular space where they could interact with soluble Aβ to facilitate its sequestration or clearance. In contrast, the high microglial colocalization in the cortex suggests that ASC specks are actively involved in the proinflammatory response surrounding mature plaques. These distinct localization patterns support the hypothesis that the functional state of ASC specks is region-dependent. This suggests that plaque formation may depend on other mechanisms, such as the preserved balance between Aβ seeding and microglial clearance.

The relative preservation of the cerebellum may be attributed to a protective feedback loop where lower Aβ deposition prevents the ‘priming’ of microglia, thereby limiting the inflammatory environment required for neurofibrillary tangle formation and spreading. Consequently, the maintenance of a non-inflammatory microglial state may act as a barrier against the toxic interaction between Aβ and tau, effectively decoupling amyloid pathology from the aggressive tauopathy and subsequent neurodegeneration seen in the cerebrum.

Distinct neuropathological responses to Aβ plaques were observed in the cerebellum and cortex of 5xFAD mice. Similarly, in AD patients, Aβ plaques in the cortex are associated with a malignant form of amyloidosis, characterized by neuritic dystrophy, neurofibrillary tangle accumulation, axonal changes, and a robust glial response. In contrast, the minimal neuropil response in the cerebellum suggests a more benign form of parenchymal amyloidosis [26]. However, the prominent atrophy in cerebral structures—such as the motor, somatosensory, and retrosplenial cortices, which are more severely affected by neurofibrillary tangles, neuritic dystrophy, and axonal changes—may secondarily lead to cerebellar atrophy, such as a reduction in Purkinje cells, during the late stages of AD [26].

The cerebellum’s resistance to plaques is also reflected in its synaptic health. While the cortex displayed dystrophic synaptic accumulations around Aβ plaques—a pathology also described in previous studies [30]—the cerebellum was spared from this significant synaptic damage. This finding is in line with previous work demonstrating that Aβ accumulation is linked to glutamatergic synapse dysfunction, particularly affecting VGLUT1-positive terminals and, to a lesser extent, VGLUT2 [31,32] in human cortical brain tissue. The almost complete absence of dystrophic VGLUT1+ neurites in the cerebellum of 5xFAD mice suggests that the cerebellum’s protective cell microenvironment not only prevents plaque formation but also preserves synaptic integrity.

Microglia, which play a central role in AD [33], appear to function in a region-specific manner. As an example of such specialization, cerebellar microglia exhibit a dynamic transcriptional and immunological profile that differs from other microglial populations [25]. Our data support a dysregulated phagocytic state of cortical microglia with dysfunctional Aβ phagocytosis. Although these cells show high levels of phagocytosis-associated markers like CD68 and are strongly associated with plaques and dystrophic synapses, they fail to fully engulf these structures. This is in line with previous studies showing that microglial phagocytic activity and cortical Aβ plaque burden exhibited a significant inverse correlation in APPPS1 mice [34]. Moreover, studies in the cortex of mouse models have found that microglia from plaque-rich areas have a different transcriptional signature than those from plaque-free regions in terms of debris clearance and phagocytosis [35,36]. In contrast, cerebellar microglia exhibit a preserved clearance capacity, despite expressing lower levels of phagocytosis-related genes such as CD68, TREM2, and CD36. This finding aligns with a growing body of evidence from epigenetic and transcriptomic studies showing that microglia in the cerebellum may express lower levels of certain phagocytosis-related genes [37,38,39] yet still maintain a highly efficient, non-inflammatory state and a high level of basal clearance activity. The unique local environment of cerebellar microglia may influence their function, enabling them to effectively clear potential threats like Aβ. For instance, the absence of Aβ dense plaque formation (even in the presence of fibrillar or diffuse Aβ accumulations) may allow microglia to maintain efficient clearance of Aβ fibrils [40]. This, in turn, would generate a positive feedback loop that prevents plaque formation in the cerebellum.

### 4.2. Potential Role of the Extracellular Matrix in Regional Resilience

Furthermore, we found that the ECM may play a novel, significant role in this regional resilience. The composition of the ECM in the cerebellum is described as being denser than in other brain regions. This is particularly evident after postnatal day 28, when the cerebellum becomes rich in brevican, a proteoglycan that plays a critical role in stabilizing synaptic connections and modulating synaptic plasticity [41]. In DCN, brevican has been shown to be expressed along with other ECM components like aggrecan, tenascin-R, and phosphacan [42,43,44].

The cerebellum of 5xFAD mice showed higher expression levels of the proteoglycans brevican and aggrecan compared to the cortex. We hypothesize that elevated ECM expression confers a protective role to synapses in the cerebellum. This is supported by recent work suggesting that increased ECM expression in the cerebellum may serve as a protective mechanism for synapses by reducing their tagging by the complement protein C3, implicated in synaptic pruning by microglia [22]. On the other hand, low Aβ burden in the cerebellum plays positive role in maintaining elevated ECM. Consistent with previous findings, Aβ accumulation has been shown to exert inhibitory effects on the synthesis and proteolytic cleavage of proteoglycans. Aβ can induce the secretion of enzymes such as MMPs and ADAMTSs [45], which are known to cleave brevican. This process leads to the marked diminution of brevican’s proteolytic fragment in the cerebral cortex and basal ganglia of AD models [46]. In the cerebellum, the low Aβ accumulation may limit the synthesis of these enzymes, thereby preserving brevican levels and safeguarding synaptic integrity. Microglia may also participate in this regulation. The finding that partial depletion of microglia in aged animals leads to increased brevican expression [47]—resembling the phenotype observed in the cerebellum of 5xFAD mice—suggests a crucial link between a non-inflammatory microglial profile and the preservation of ECM components.

We also observed enhanced internalization of brevican alongside small Aβ aggregates by cerebellar microglia, a process that was less prominent in cortical microglia. These findings suggest that the unique interactions between microglia and the ECM—specifically the efficient uptake and degradation of ECM-Aβ co-aggregates—can be a key mechanism underlying the cerebellum’s resilience to amyloid pathology. This hypothesis is supported by previous studies showing that proteoglycans can bind directly to both fibrillar and non-fibrillar Aβ, influencing its aggregation, stability, and proteolytic degradation [48,49]. By sequestering Aβ in a non-fibrillar form, the ECM-Aβ complexes are less neurotoxic to neurons than the aggregated Aβ alone. This interaction with ECM may change the mechanisms of uptake and trafficking of Aβ within cells, particularly in microglia.

Our findings suggest that ECM components, such as brevican, may have a high affinity for non-fibrillar or early-stage Aβ in the cerebellum, potentially facilitating Aβ sequestration. In contrast, ECM modifications in the cortex—possibly driven by proinflammatory microglial MMPs and ADAMTSs that cleave brevican and aggrecan or different pattern of glycosylation—may at least partially explain the reduced Aβ sequestration in this region. Therefore, further studies on the cascade of ECM degradation in both regions are essential to understanding these regional differences.

As tau interacts with ECM proteins [50], we can expect that differences between cerebellar and cortical ECM and microglia states may also result in differences in the formation and spreading of neurofibrillary tangles in AD patients. However, the 5xFAD mouse line does not develop tangles [19] and is therefore not an appropriate model for this question.

Apart from being expressed in PNNs, brevican is the major component of perisynaptic and interstitial ECM [51]. It may form complexes with Aβ and other ECM molecules, which could be more efficiently internalized and degraded by microglia, perivascular macrophages, and astrocytes, or more efficiently transported into the perivascular space for clearance via the glymphatic system. Further studies should elucidate these multiple possible mechanisms and eventually lead to the development of new therapeutic strategies targeting the ECM in AD.

Our findings suggest a complex scenario involving brevican and possibly other ECM molecules, such as laminin, which may contribute to the inhibition of Aβ fibril formation and to the internalization of Aβ by microglia. In this context, it is noteworthy that laminin undergoes distinct, region-specific changes with age. Studies have shown that while laminin levels decrease in the aging cortex, they are conserved in the cerebellum [52]. Further investigation is warranted to fully elucidate the relationship between laminin and brevican in the context of AD.

## 5. Conclusions

Our findings point to a neuroprotective role of ECM in the cerebellum; it actively interacts with toxic Aβ proteins, which facilitate their rapid internalization by microglia and prevent plaque formation, thereby protecting synaptic integrity. To build on our findings, subsequent studies are needed to dissect the precise ECM-mediated mechanisms in the cerebellum in the context of AD. A key question is how microglia utilize a combination of proteolytic enzyme secretion with endocytic and phagocytic internalization to efficiently deal with ECM-Aβ aggregates.

Understanding these mechanisms could provide new insights into AD pathogenesis and help identify promising therapeutic targets for brain regions heavily affected by amyloidosis. Therefore, therapeutic strategies aimed at modulating the ECM-microglia interface—either by enhancing the expression of specific Aβ42 clearance-related proteoglycans or by promoting microglial uptake of ECM-Aβ co-aggregates—may provide a novel approach to inhibiting microglial inflammatory mechanisms and preventing dense plaque formation and associated synaptic loss.

## Data Availability

Data supporting reported results can be obtained upon request to the corresponding author (A.D.).

## Supplementary Materials

The following supporting information can be downloaded at https://www.mdpi.com/article/10.3390/cells15020182/s1. Figure S1: Colocalization between brevican and Aβ.
