# Supplementary figures and images for: Cerebellar Resistance to Amyloid Plaque Deposition and Elevated Microglial ECM Proteoglycan Uptake in 5xFAD Mice
Source: Cells. 2026 Jan 19;15(2):182. doi: 10.3390/cells15020182 (PMC12839178; doi:10.3390/cells15020182)

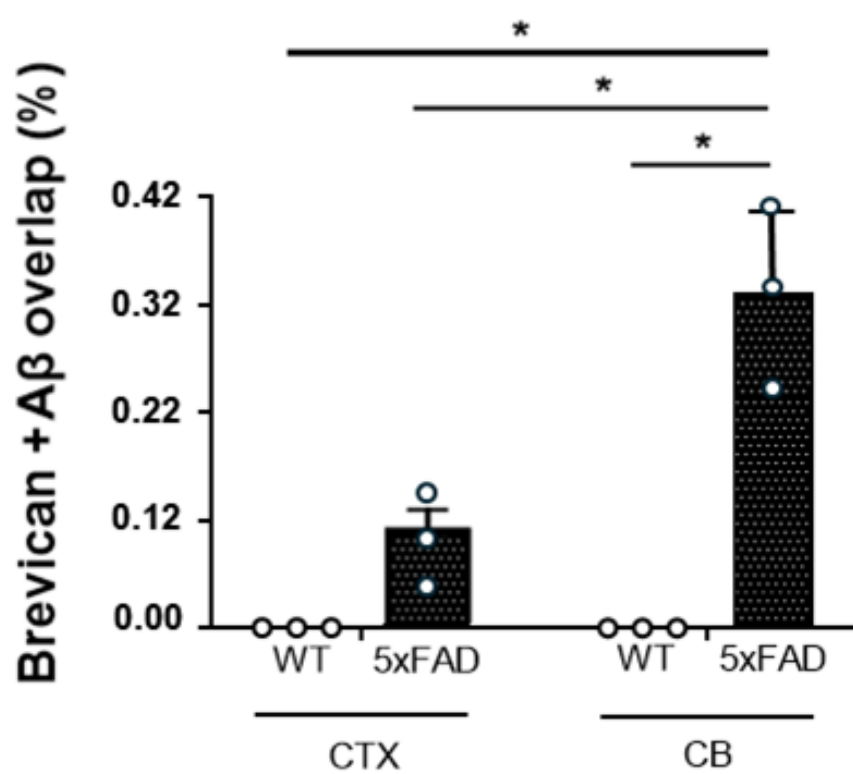

Figure S1. Colocalization between Brevican and A $\beta$ .

Supplement: Supplementary file 1 [file cells-15-00182-s001.zip › cells-4077103-supplementary.pdf]
